# Rapid West Nile Virus Antigen Detection

**DOI:** 10.3201/eid1110.040394

**Published:** 2005-10

**Authors:** Nicholas A. Panella, Kristen L. Burkhalter, Stanley A. Langevin, Aaron C. Brault, Lynn M. Schooley, Brad J. Biggerstaff, Roger S. Nasci, Nicholas Komar

**Affiliations:** * Centers for Disease Control and Prevention, Fort Collins, Colorado, USA

**Keywords:** West Nile virus, flavivirus, oral swab, cloacal swab, arbovirus surveillance, dispatch

## Abstract

We compared the VecTest WNV antigen assay with standard methods of West Nile virus (WNV) detection in swabs from American Crows (*Corvus brachyrhynchos*) and House Sparrows (*Passer domesticus*). The VecTest detected WNV more frequently than the plaque assay and was comparable to a TaqMan reverse transcription–polymerase chain reaction.

Dead bird surveillance is an effective way to monitor the presence and spread of West Nile virus (WNV) in North America (*1*), and assays to detect infectious WNV virions, antigen, and RNA in tissues from infected birds are reliable techniques (*2**–**4*). More than 28,000 bird carcasses were tested for WNV in the United States from 1999 to 2002 (*5*). Processing and testing these carcasses require a substantial commitment of resources from federal, state, and local health departments. Simplifying diagnostic procedures by implementing rapid antigen-capture assays would permit increased specimen processing and, ultimately, improved surveillance.

Cloacal and oral (nasopharyngeal) swabs from dead corvids (crows and jays) are reliable sources of WNV RNA and infectious virions (*6*). Three field evaluations of an antigen detection assay applied to corvid carcasses collected shortly after death found that oral swabs were more sensitive than cloacal swabs for detecting WNV antigen, and that sensitivity of the VecTest WNV antigen assay (Medical Analysis Systems, Camarillo, CA, USA) applied to oral swabs was >80% for American Crows, lower for other corvids, and variable for a variety of other species (*7**–**9*). Several questions remain unanswered regarding the usefulness of swab specimens for WNV surveillance. How long after death of a bird can WNV be detected in swab specimens? Can swabs from noncorvid birds be used to detect WNV? Can reverse transcription–polymerase chain reaction (RT-PCR) or VecTest detect WNV in oral swab samples that have remained dry and at room temperature?

To address these questions, we compared the VecTest WNV antigen assay with standard methods of virus detection from oral and cloacal swabs taken 1–4 days postmortem from experimentally infected American Crows (*Corvus brachyrhynchos*) and House Sparrows (*Passer domesticus*). The VecTest, which was originally developed for mosquito pools as a simple, 1-step wicking assay available in a kit, requires no specialized equipment, storage conditions, or highly trained personnel and provides results in 15 minutes (*10**,**11*).

## The Study

Oral and cloacal swab samples were collected daily (for 4 days) from carcasses of crows and sparrows that had been experimentally infected with either the NY99-4132 strain (30 crows and 6 sparrows) or the Kenyan KN-3829 strain (1 crow and 5 sparrows) of WNV. Carcasses were stored at ambient temperature (≈20°C) during this period. The samples were collected with standard, cotton-tipped applicators by inserting them into the cloaca or oral cavity and then placing them directly into 1 mL VecTest grinding solution A, a physiologic buffer similar to phosphate-buffered saline. Samples were subsequently frozen at –70°C until tested by a variety of methods for detecting WNV. Some oral swabs from infected crows were left at room temperature without diluent for 24 or 48 hours before testing. Negative control swab samples were collected from 25 live, healthy, uninfected crows.

All swab specimens collected from crow carcasses were positive by the TaqMan RT-PCR method, using 2 sets of WNV-specific primers (*2*). Several TaqMan RT-PCR–negative swabs for the sparrows were also negative by the other assays; these were disregarded in summarizing the results. Results of VecTest and Vero plaque assay (*11*) of the RT-PCR–positive swab specimens are shown as sensitivities (using RT-PCR as the standard for detecting WNV RNA) in Table 1. A logistic regression model accounting for anticipated correlation induced by multiple and repeated observations on each bird was used to compare sensitivities for each day postmortem, with significance determined using α = 0.05 (*12*). For crows evaluated 1 day postmortem, no significant difference between swab types (oral versus cloacal) (p = 0.63) and no significant difference between the 2 assays (p = 0.10) were detected. At 2 days postmortem, the effect due to swab type was not significant (p = 0.07), but a significant difference was seen in the sensitivities of the 2 assays (p = 0.004), excluding the nonsignificant effect of swab type from the logistic regression model. At 3 days postmortem, both swab type and assay differences were significant (p<0.01), with oral swabs more likely to yield a positive finding (compared with cloacal swabs) and VecTest more sensitive than plaque assay. For sparrows, no significant differences were seen between the sensitivities of the VecTest and plaque assay for either swab type on any of the 3 days (McNemar test).

**Table 1:** Sensitivities of the VecTest and plaque assay in detecting West Nile virus in American Crows and House Sparrows, by swab type and day postmortem*

Species	Day postmortem	Assay	Swab type	
			Oral, % positive (no. tested)	Cloacal, % positive (no. tested)
American Crow	1	VecTest	90 (30)	90 (29)
		Plaque	83 (30)	69 (29)
	2	VecTest	93 (30)	100 (29)
		Plaque	90 (30)	55 (29)
	3	VecTest	97 (30)	83 (30)
		Plaque	83 (30)	13 (30)
	4	VecTest	100 (5)	80 (5)
		Plaque	80 (5)	0 (5)
House Sparrow	1	VecTest	70 (10)	67 (9)
		Plaque	90 (10)	56 (9)
	2	VecTest	75 (8)	100 (2)
		Plaque	75 (8)	50 (2)
	3	VecTest	83 (6)	NA
		Plaque	83 (6)	NA

*NA, not available (valid sampling was not possible given the physical state of the bird).

VecTest detected WNV in 90% of 22 crow oral swabs that were tested after remaining dry and at room temperature for 24 hours and in 70% of 13 crow oral swabs assayed after 48 hours. By comparison, TaqMan RT-PCR detected WNV in 86% and 70% of these oral swabs, respectively, at the same time points.

Over the 4-day sampling period, geometric mean viral titers in crow oral swabs, determined by Vero cell plaque assay, decreased from 10^3.6^ to 10^2.2^ PFU/swab (Table 2). In contrast, the geometric mean viral titer in crow cloacal swabs decreased from 10^3.0^ PFU/swab at 1 day postmortem to undetectable by 4 days postmortem. RNA levels, as detected by the TaqMan assay, also decreased over time.

**Table 2:** Log geometric mean titer (SD) of West Nile virus PFU equivalents (TaqMan RT-PCR) and PFU (plaque assay) for American Crows and House Sparrows, by swab type and day postmortem*

Species	Swab type	Assay	Day postmortem			
			1	2	3	4
American Crow	Oral	TaqMan	4.8 (1.1)	4.7 (0.6)	4.3 (0.8)	3.5 (1.5)
		Plaque	3.6 (1.9)	3.2 (1.5)	2.4 (1.6)	2.2 (2.0)
	Cloacal	TaqMan	5.2 (1.2)	5.5 (1.3)	4.6 (1.9)	3.5 (0.7)
		Plaque	3.0 (2.4)	1.4 (1.5)	0.3 (0.7)	0.0 (0.0)
House Sparrow	Oral	TaqMan	4.3 (1.4)	5.0 (1.7)	4.6 (1.7)	NA
		Plaque	3.8 (2.0)	3.0 (1.9)	2.9 (2.5)	NA
	Cloacal	TaqMan	3.8 (1.6)	4.3 (3.3)	NA	NA
		Plaque	1.9 (2.9)	0.7 (0.9)	NA	NA

*RT-PCR, reverse transcription–polymerase chain reaction; NA, not available (valid sampling was not possible given the physical state of the bird).

## Conclusions

VecTest has the potential to simplify dead bird surveillance for WNV by reducing required resources such as specialized equipment and costly reagent kits needed to achieve a rapid and accurate result. With appropriate biosafety measures, the assay can be conducted in the field, or in centralized regional laboratories, obviating the need for expensive shipping of bird carcasses to remote reference laboratories.

One objective of our study was to determine whether oral or cloacal swabs were preferable for WNV testing of dead birds. To answer this question, several criteria were evaluated, including the ability of 3 different assays to detect WNV, the feasibility of collecting specimens postmortem, and postmortem duration of WNV positivity. TaqMan RT-PCR detected WNV RNA and antigen in similar proportions in all cloacal and oral specimens collected from crows. However, virus isolation by Vero plaque assay was more successful when oral swabs were tested. Virus appears to be more rapidly inactivated in the cloaca compared with the oral cavity. This phenomenon was consistent for both sparrows and crows.

Fewer postmortem swab samples were available from sparrows compared with those from crows because fewer sparrow carcasses were available (sparrows are less susceptible to fatal WNV infection than crows) (*13*). Collecting cloacal swabs from the smaller sparrows was also more difficult after 1 day postmortem because they tended to desiccate quickly. RT-PCR detected WNV RNA in sparrows from 24/24 oral swabs, but only 11/13 cloacal swabs. Antigen was detected by VecTest from 18/24 oral and 8/13 cloacal swabs. Infectious virus was detected by plaque assay in 20/24 oral swabs but in only 6/13 cloacal swabs. Virus titers and RNA concentrations in the carcasses decayed over the 4-day period of observation and this decay was most pronounced in the cloacal swabs. Thus, oral swabs were more effective than cloacal swabs to detect WNV in both crows and sparrows.

VecTest consistently detected WNV antigen in a greater proportion of samples than Vero plaque assay detected virions. Thus, although the detection of infectious virus was inconsistent, carcasses contained sufficient quantity of viral components, both RNA and protein, to permit detection for >4 days after death. In a natural setting, carcasses most likely would decay more rapidly than in these experiments, given exposure to temperature fluctuations, microbial attack, and predation. Guidelines for WNV surveillance recommend sampling carcasses <24 hours old (*14*). These results suggest that older carcasses may have detectable WNV RNA and antigen that still are readily detectable with the TaqMan and VecTest assays. Thus, carcasses should be tested regardless of age, as long as they are not in a condition where sampling is impossible. In addition, swabs collected in the field can be stored at room temperature in empty cryovials for up to 48 hours and then reliably assayed for WNV antigen by VecTest.

Detecting WNV from sparrow carcasses demonstrates that swabs are useful to test species other than corvids. House sparrows, like corvids, are passerine birds that develop high levels of WNV in blood and tissues (*13*). Stone et al. showed that the VecTest had a sensitivity of 76% in detecting WNV in oral swabs of field-collected carcasses of house sparrows (*9*).

In summary, oral swabs are more useful than cloacal swabs for obtaining a reliable result with the diagnostic assays described in our study. Moreover, swabs from noncorvid birds may also be effectively assayed for WNV. Our findings suggest that large numbers of dead corvids of any age, and possibly other passerine birds, could be screened by cautiously collecting dry oral swabs in the field, storing them properly, and then testing them within 48 hours by rapid antigen detection assay or RT-PCR.
